# The genome sequence of the Sandy Carpet moth, *Perizoma flavofasciatum *(Thunberg, 1792)

**DOI:** 10.12688/wellcomeopenres.23612.1

**Published:** 2025-02-03

**Authors:** Douglas Boyes, Jonathan Davis, University of Oxford and Wytham Woods Genome Acquisition Lab, Wellcome Sanger Institute Tree of Life Management, Samples and Laboratory team, Wellcome Sanger Institute Scientific Operations: Sequencing Operations, Wellcome Sanger Institute Tree of Life Core Informatics team, Tree of Life Core Informatics collective, Darwin Tree of Life Consortium

**Affiliations:** 1 UK Centre for Ecology & Hydrology, Wallingford, England, UK; 2 Independent researcher, Lanark, Scotland, UK

**Keywords:** Perizoma flavofasciatum, Sandy Carpet moth, genome sequence, chromosomal, Lepidoptera

## Abstract

We present a genome assembly from a male specimen of *Perizoma flavofasciatum* (Sandy Carpet; Arthropoda; Insecta; Lepidoptera; Geometridae). The genome sequence has a total length of 369.30 megabases. Most of the assembly (99.88%) is scaffolded into 30 chromosomal pseudomolecules, including the Z sex chromosome. The mitochondrial genome has also been assembled and is 16.61 kilobases in length. Gene annotation of this assembly on Ensembl identified 11,915 protein-coding genes.

## Species taxonomy

Eukaryota; Opisthokonta; Metazoa; Eumetazoa; Bilateria; Protostomia; Ecdysozoa; Panarthropoda; Arthropoda; Mandibulata; Pancrustacea; Hexapoda; Insecta; Dicondylia; Pterygota; Neoptera; Endopterygota; Amphiesmenoptera; Lepidoptera; Glossata; Neolepidoptera; Heteroneura; Ditrysia; Obtectomera; Geometroidea; Geometridae; Larentiinae; *Perizoma*; *Perizoma flavofasciatum* (Thunberg, 1792) (NCBI:txid934819)

## Background

*Perizoma flavofasciatum* (family Geometridae), commonly known as the Sandy Carpet moth, is widely distributed across Europe and extends into parts of Asia. Its range includes most European countries and stretches eastwards through the Palaearctic region to the Urals and the Altai Mountains ( GBIF Secretariat, 2023). This species inhabits various environments across its range, such as meadow valleys, floodplains, waterside areas, bushy meadows, and gardens. In mountainous regions like the Alps, it can be found at elevations up to 1,500 metres.

*Perizoma flavofasciatum* has a wingspan of 26–32 mm ( Kimber, 2025). The species is characterised by the sandy brown cross-lines on a white ground colour and the two interneural blotches connecting the median and subterminal fasciae at about halfway between costa and dorsum ( British Lepidoptera, 2025).

In the UK, it is fairly common across Britain, particularly in the south of England ( GBIF Secretariat, 2023) and is listed as “least concern” in the macro-moth status review ( Fox *et al.*, 2019). The adult flies from dusk onwards in June and July ( Kimber, 2025), and inhabits woodland, commons, chalky ground and other dry areas. The larvae feed on the seed-pods of campions ( *Silene spp.*) ( Waring *et al.*, 2017).

**Figure 1.**
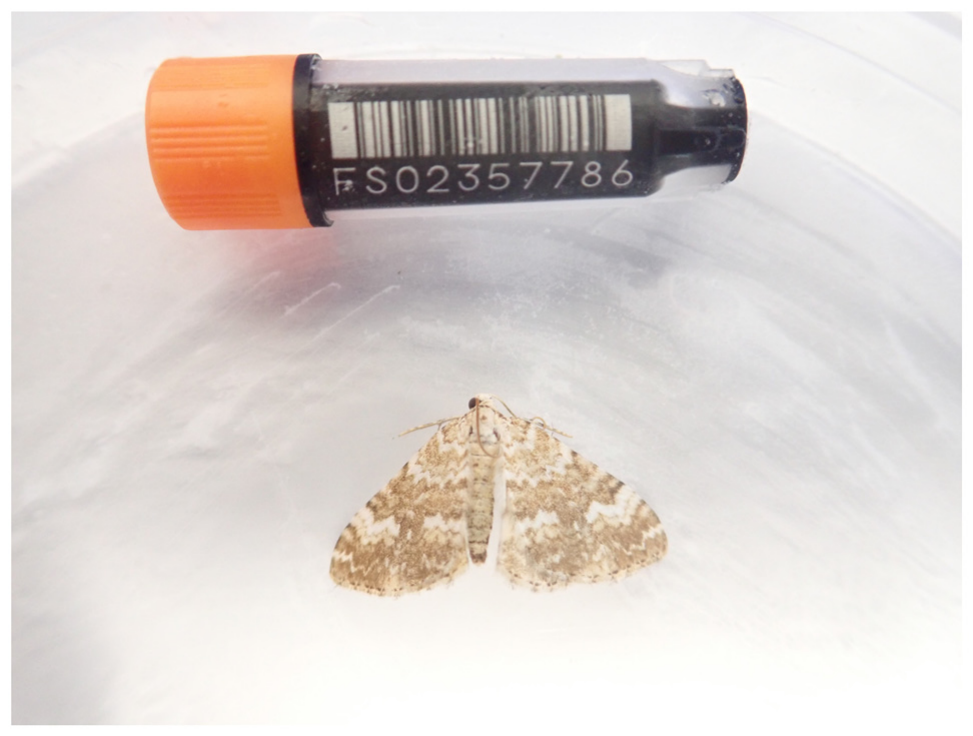
Photograph of the *Perizoma flavofasciatum* (ilPerFlao1) specimen used for genome sequencing.

We present a chromosomal-level genome sequence for *Perizoma flavofasciatum*, based on a male specimen from Wytham Woods, Berkshire, United Kingdom ( Figure 1). This was sequenced as part of the Darwin Tree of Life project.

## Genome sequence report

The genome of *Perizoma flavofasciatum* ( Figure 1) was sequenced using Pacific Biosciences single-molecule HiFi long reads, generating a total of 25.84 Gb (gigabases) from 2.00 million reads, providing an estimated 66-fold coverage. Primary assembly contigs were scaffolded with chromosome conformation Hi-C data, which produced 91.65 Gb from 606.97 million reads. Specimen and sequencing details are summarised in Table 1.

**Table 1.** Specimen and sequencing data for *Perizoma flavofasciatum*.

Project information			
**Study title**	Perizoma flavofasciatum (sandy carpet)		
**Umbrella BioProject**	PRJEB63430		
**Species**	*Perizoma flavofasciatum*		
**BioSample**	SAMEA7701445		
**NCBI taxonomy ID**	934819		
Specimen information			
**Technology**	**ToLID**	**BioSample accession**	**Organism part**
**PacBio long read** **sequencing**	ilPerFlao1	SAMEA7701609	Whole organism
**Hi-C sequencing**	ilPerFlao2	SAMEA112360821	thorax
**RNA sequencing**	ilPerFlao2	SAMEA112360822	abdomen
Sequencing information			
**Platform**	**Run accession**	**Read count**	**Base count (Gb)**
**Hi-C Illumina NovaSeq** **6000**	ERR11606315	6.07e+08	91.65
**PacBio Sequel IIe**	ERR11593799	2.00e+06	25.84
**RNA Illumina NovaSeq** **6000**	ERR11837494	7.68e+07	11.59

Assembly errors were corrected by manual curation, including three missing joins or mis-joins. The final assembly has a total length of 369.30 Mb in 39 sequence scaffolds, with three gaps. The scaffold N50 is 13.4 Mb ( Table 2).

**Table 2.** Genome assembly data for *Perizoma flavofasciatum*, ilPerFlao1.1.

Genome assembly		
Assembly name	ilPerFlao1.1	
Assembly accession	GCA_958496245.1	
*Accession of alternate haplotype*	*GCA_958496225.1*	
Span (Mb)	369.30	
Number of contigs	43	
Number of scaffolds	39	
Longest scaffold (Mb)	20.74	
Assembly metrics *		*Benchmark*
Contig N50 length (Mb)	13.4	*≥ 1 Mb*
Scaffold N50 length (Mb)	13.4	*= chromosome N50*
Consensus quality (QV)	65.5	*≥ 40*
*k*-mer completeness	Primary: 88.65%; alternate: 82.01%; combined: 99.57%	*≥ 95%*
BUSCO v5.4.3 lineage: lepidoptera_odb10	C:98.2%[S:97.8%,D:0.4%], F:0.4%,M:1.4%,n:5,286	*S > 90%*, *D < 5%*
Percentage of assembly mapped to chromosomes	99.88%	*≥ 90%*
Sex chromosomes	Z	*localised homologous* *pairs*
Organelles	Mitochondrial genome: 16.61 kb	*complete single alleles*
Genome annotation of assembly GCA_958496245.1 at Ensembl		
Number of protein-coding genes	11,915	
Number of non-coding genes	1,605	
Number of gene transcripts	22,268	

* Assembly metric benchmarks are adapted from Rhie *et al.* (2021) and the Earth BioGenome Project Report on Assembly Standards September 2024. ** BUSCO scores based on the lepidoptera_odb10 BUSCO set using version 5.4.3. C = complete [S = single copy, D = duplicated], F = fragmented, M = missing, n = number of orthologues in comparison.

**Figure 2.**
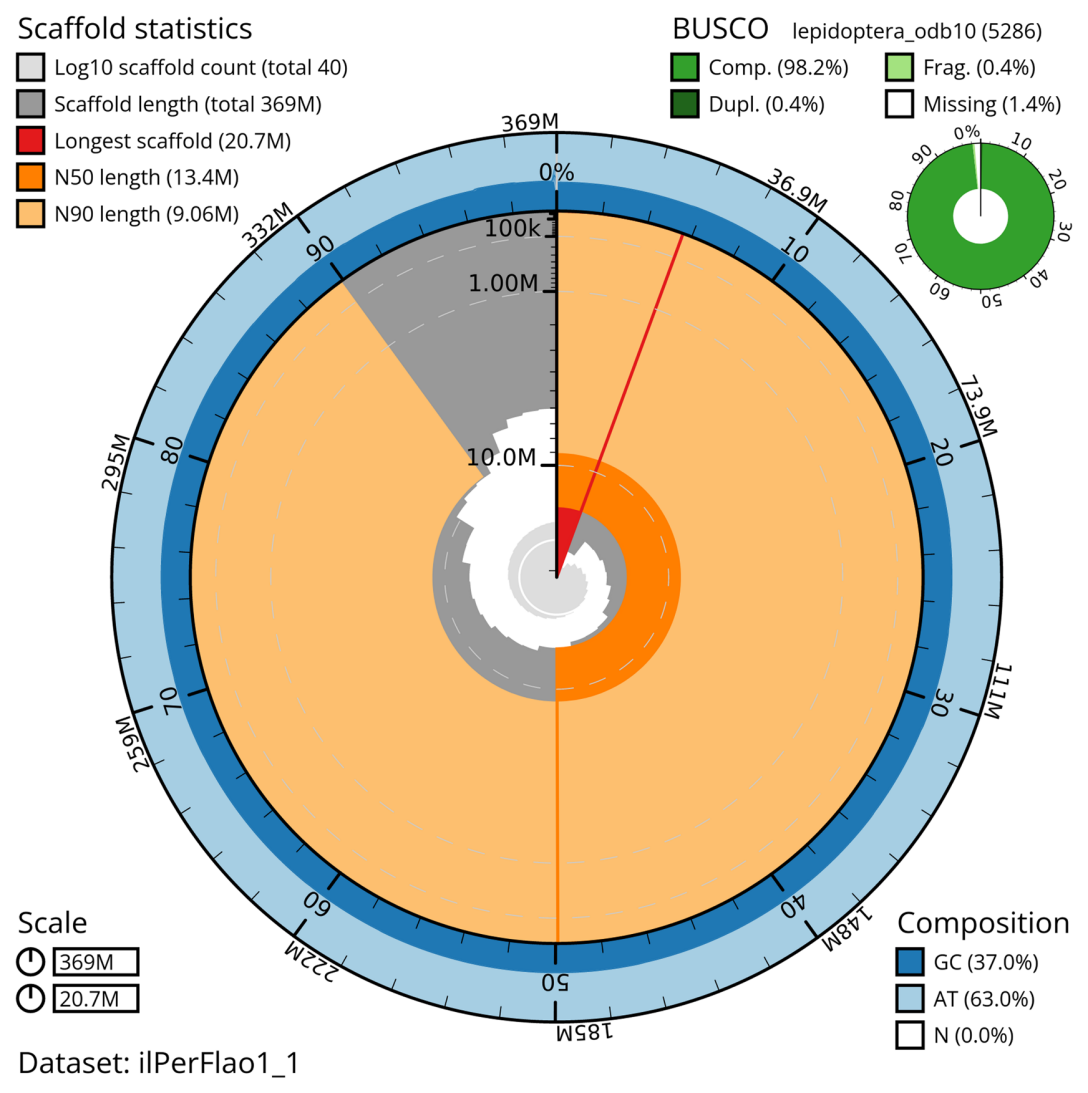
Genome assembly of *Perizoma flavofasciatum*, ilPerFlao1.1: metrics. The BlobToolKit snail plot provides an overview of assembly metrics and BUSCO gene completeness. The circumference represents the length of the whole genome sequence, and the main plot is divided into 1,000 bins around the circumference. The outermost blue tracks display the distribution of GC, AT, and N percentages across the bins. Scaffolds are arranged clockwise from longest to shortest and are depicted in dark grey. The longest scaffold is indicated by the red arc, and the deeper orange and pale orange arcs represent the N50 and N90 lengths. A light grey spiral at the centre shows the cumulative scaffold count on a logarithmic scale. A summary of complete, fragmented, duplicated, and missing BUSCO genes in the lepidoptera_odb10 set is presented at the top right. An interactive version of this figure is available at https://blobtoolkit.genomehubs.org/view/ilPerFlao1_1/dataset/ilPerFlao1_1/snail.

**Figure 3.**
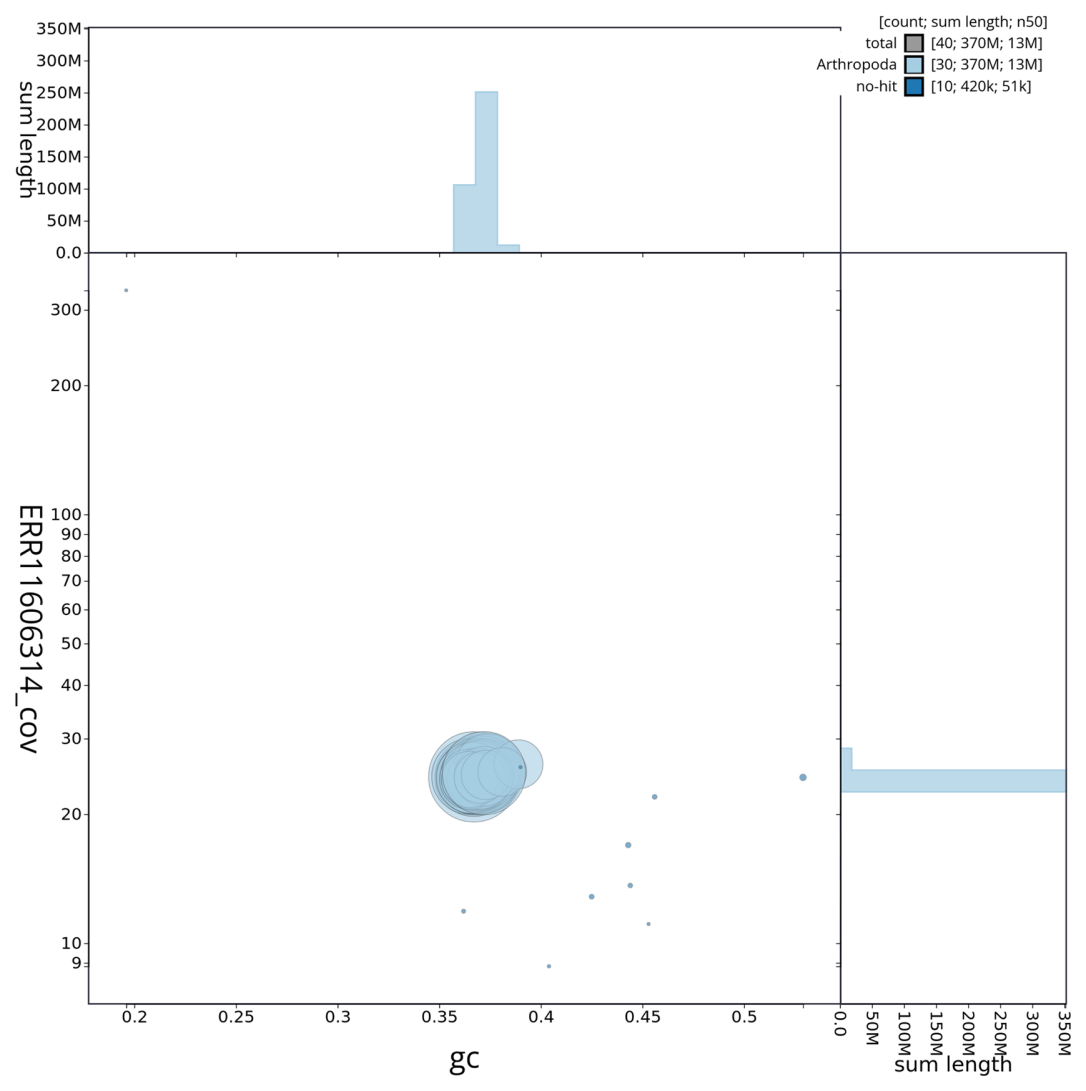
Genome assembly of *Perizoma flavofasciatum*, ilPerFlao1.1: BlobToolKit GC-coverage plot showing sequence coverage (vertical axis) and GC content (horizontal axis). The circles represent scaffolds, with the size proportional to scaffold length and the colour representing phylum membership. The histograms along the axes display the total length of sequences distributed across different levels of coverage and GC content. An interactive version of this figure is available at https://blobtoolkit.genomehubs.org/view/ilPerFlao1_1/dataset/ilPerFlao1_1/blob.

**Figure 4.**
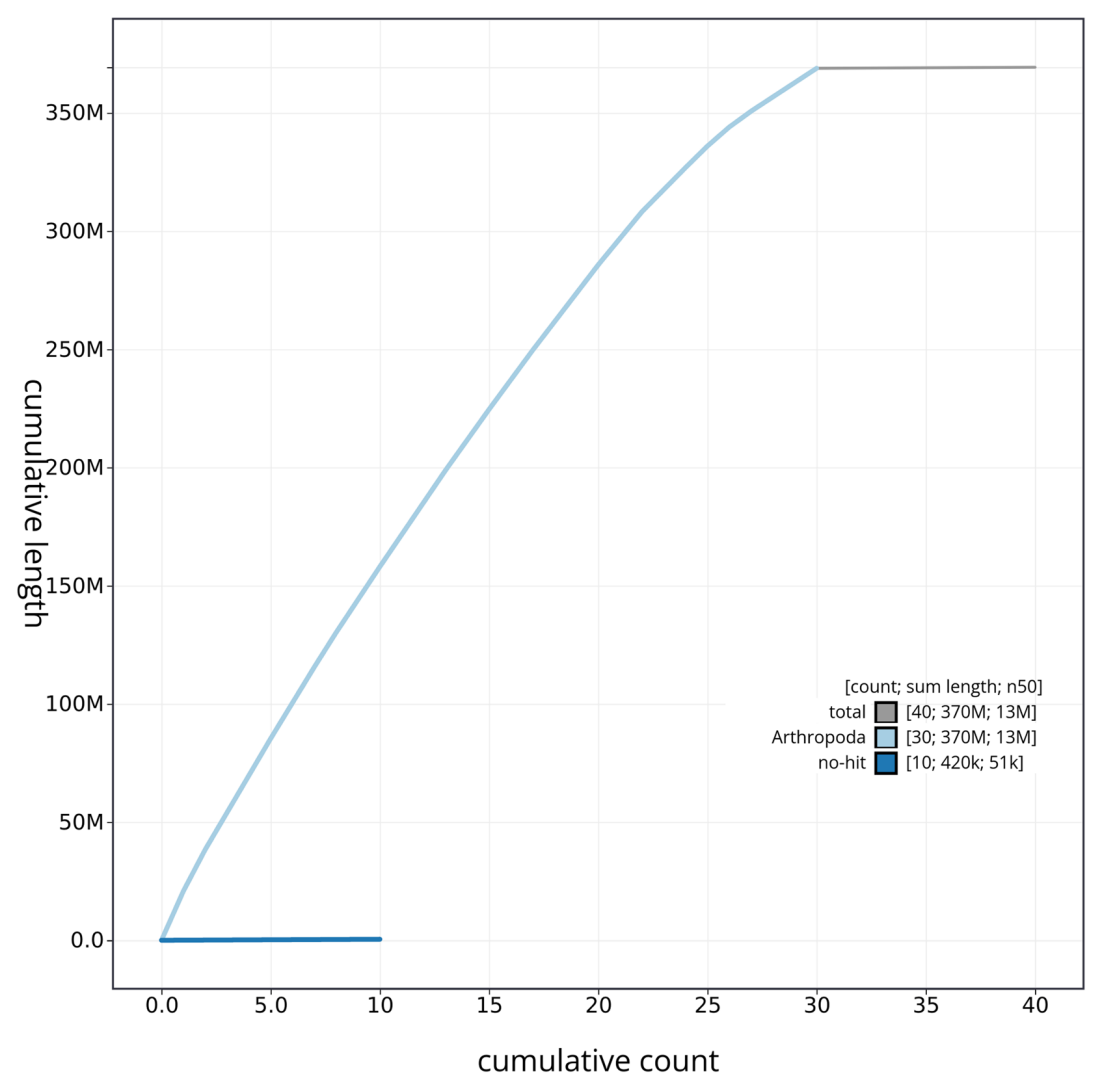
Genome assembly of *Perizoma flavofasciatum* ilPerFlao1.1: BlobToolKit cumulative sequence plot. The grey line shows cumulative length for all scaffolds. Coloured lines show cumulative lengths of scaffolds assigned to each phylum using the buscogenes taxrule. An interactive version of this figure is available at https://blobtoolkit.genomehubs.org/view/ilPerFlao1_1/dataset/ilPerFlao1_1/cumulative.

The snail plot in Figure 2 provides a summary of the assembly statistics, indicating the distribution of scaffold lengths and other assembly metrics. Figure 3 shows the distribution of scaffolds by GC proportion and coverage. Figure 4 presents a cumulative assembly plot, with separate curves representing different scaffold subsets assigned to various phyla, illustrating the completeness of the assembly.

**Figure 5.**
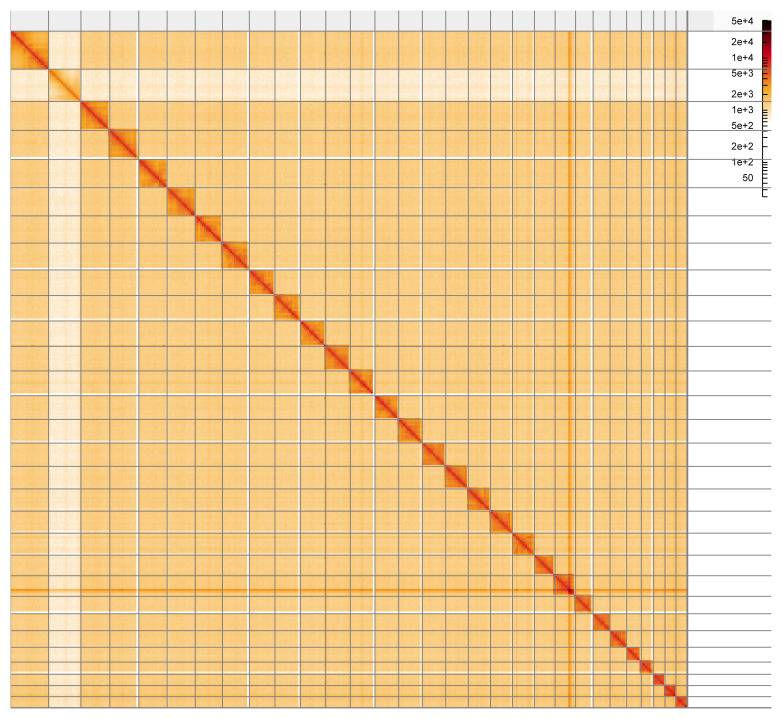
Genome assembly of *Perizoma flavofasciatum* ilPerFlao1.1: Hi-C contact map of the ilPerFlao1.1 assembly, visualised using HiGlass. Chromosomes are shown in order of size from left to right and top to bottom. An interactive version of this figure may be viewed at https://genome-note-higlass.tol.sanger.ac.uk/l/?d=KlJLbAflRRWP4E1ra_QFAQ.

Most of the assembly sequence (99.88%) was assigned to 30 chromosomal-level scaffolds, representing 29 autosomes and the Z sex chromosome. These chromosome-level scaffolds, confirmed by the Hi-C data, are named in order of size ( Figure 5; Table 3). During manual curation the Z chromosome was identified based on synteny with *Eulithis prunata* (GCA_918843925.1) ( Boyes *et al.*, 2023).

**Table 3.** Chromosomal pseudomolecules in the genome assembly of *Perizoma flavofasciatum*, ilPerFlao1.

INSDC accession	Name	Length (Mb)	GC%
OY292419.1	1	20.74	36.5
OY292421.1	2	15.81	37.0
OY292422.1	3	15.75	37.0
OY292423.1	4	15.46	37.0
OY292424.1	5	15.35	36.5
OY292425.1	6	14.89	37.0
OY292426.1	7	14.63	36.5
OY292427.1	8	13.98	37.0
OY292428.1	9	13.86	37.0
OY292429.1	10	13.83	36.5
OY292430.1	11	13.42	37.0
OY292431.1	12	13.42	37.0
OY292432.1	13	12.96	37.0
OY292433.1	14	12.9	36.5
OY292434.1	15	12.72	37.0
OY292435.1	16	12.32	37.0
OY292436.1	17	12.15	37.0
OY292437.1	18	11.99	37.0
OY292438.1	19	11.94	37.0
OY292439.1	20	11.24	36.5
OY292440.1	21	11.2	37.5
OY292441.1	22	9.54	37.0
OY292442.1	23	9.2	36.5
OY292443.1	24	9.06	37.0
OY292444.1	25	8.06	36.5
OY292445.1	26	6.7	37.0
OY292446.1	27	6.17	37.5
OY292447.1	28	6.03	39.0
OY292448.1	29	5.97	38.0
OY292420.1	Z	17.6	37.0
OY292449.1	MT	0.02	19.5

While not fully phased, the assembly deposited is of one haplotype. Contigs corresponding to the second haplotype have also been deposited.

The mitochondrial genome was also assembled and can be found as a contig within the multifasta file of the genome submission, and as a separate fasta file.

The final assembly has a Quality Value (QV) of 65.5. The *k*-mer completeness value for the primary assembly was estimated as 88.65%, for the alternate haplotype was 82.01%, and of the combined assemblies was 99.57%. BUSCO (v5.4.3) analysis using the lepidoptera_odb10 reference set ( *n* = 5,286) indicated a completeness score of 98.2% (single = 97.8%, duplicated = 0.4%). The assembly achieves the EBP reference standard of 6.7.65. Other quality metrics are given in Table 2. 

## Genome annotation report

The *Perizoma flavofasciatum* genome assembly (GCA_958496245.1) was annotated at the European Bioinformatics Institute (EBI) on Ensembl Rapid Release. The resulting annotation includes 22,268 transcribed mRNAs from 11,915 protein-coding and 1,605 non-coding genes ( Table 2; https://rapid.ensembl.org/Perizoma_flavofasciatum_GCA_958496245.1/Info/Index). The average transcript length is 14,574.44. There are 1.65 coding transcripts per gene and 7.73 exons per transcript.

## Methods

### Sample acquisition and DNA barcoding

An adult male specimen of *Perizoma flavofasciatum* (specimen ID Ox000581, ToLID ilPerFlao1) was collected from Wytham Woods, Berkshire, United Kingdom (latitude 51.77, longitude –1.34) on 2020-07-05, using a light trap. The specimen was collected and identified by Douglas Boyes (University of Oxford) and on dry ice.

The specimen used for Hi-C and RNA sequencing (specimen ID SAN00002560, ToLID ilPerFlao2) was collected from Little Sparta, Dunsyre, Pentland Hills, South Lanarkshire, Scotland (latitude 55.72, longitude –3.50) on 2022-06-17. The specimen was collected and identified by Jo Davis (independent researcher).

### Nucleic acid extraction

The workflow for high molecular weight (HMW) DNA extraction at the Wellcome Sanger Institute (WSI) Tree of Life Core Laboratory includes a sequence of procedures: sample preparation and homogenisation, DNA extraction, fragmentation and purification. Detailed protocols are available on protocols.io ( Denton *et al.*, 2023b). The ilPerFlao1 sample was prepared for DNA extraction by weighing and dissecting it on dry ice ( Jay *et al.*, 2023). Tissue from the whole organism was homogenised using a PowerMasher II tissue disruptor ( Denton *et al.*, 2023a). HMW DNA was extracted using the Automated MagAttract v1 protocol ( Sheerin *et al.*, 2023). DNA was sheared into an average fragment size of 12–20 kb in a Megaruptor 3 system ( Todorovic *et al.*, 2023). Sheared DNA was purified by solid-phase reversible immobilisation, using AMPure PB beads to eliminate shorter fragments and concentrate the DNA ( Strickland *et al.*, 2023). The concentration of the sheared and purified DNA was assessed using a Nanodrop spectrophotometer and a Qubit Fluorometer using the Qubit dsDNA High Sensitivity Assay kit. The fragment size distribution was evaluated by running the sample on the FemtoPulse system.

RNA was extracted from abdomen tissue of ilPerFlao2 in the Tree of Life Laboratory at the WSI using the RNA Extraction: Automated MagMax™ *mir*Vana protocol ( do Amaral *et al.*, 2023). The RNA concentration was assessed using a Nanodrop spectrophotometer and a Qubit Fluorometer using the Qubit RNA Broad-Range Assay kit. Analysis of the integrity of the RNA was done using the Agilent RNA 6000 Pico Kit and Eukaryotic Total RNA assay.

### Hi-C preparation

Thorax tissue of the ilPerFlao2 sample was processed at the WSI Scientific Operations core, using the Arima-HiC v2 kit. Tissue (stored at –80 °C) was fixed, and the DNA crosslinked using a TC buffer with 22% formaldehyde. After crosslinking, the tissue was homogenised using the Diagnocine Power Masher-II and BioMasher-II tubes and pestles. Following the kit manufacturer's instructions, crosslinked DNA was digested using a restriction enzyme master mix. The 5’-overhangs were then filled in and labelled with biotinylated nucleotides and proximally ligated. An overnight incubation was carried out for enzymes to digest remaining proteins and for crosslinks to reverse. A clean up was performed with SPRIselect beads prior to library preparation.

### Library preparation and sequencing

***PacBio HiFi***

Libraries were prepared using the PacBio Express Template Preparation Kit v2.0 (Pacific Biosciences, California, USA) as per the manufacturer's instructions. The kit includes the reagents required for removal of single-strand overhangs, DNA damage repair, end repair/A-tailing, adapter ligation, and nuclease treatment. Library preparation also included a library purification step using AMPure PB beads (Pacific Biosciences, California, USA) and size selection step to remove templates shorter than 3 kb using AMPure PB modified SPRI. DNA concentration was quantified using the Qubit Fluorometer v2.0 (Thermo Fisher Scientific) and Qubit HS Assay Kit and the final library fragment size analysis was carried out using the Agilent Femto Pulse Automated Pulsed Field CE Instrument (Agilent Technologies).

Samples were sequenced using the Sequel IIe system (Pacific Biosciences, California, USA). The concentration of the library loaded onto the Sequel IIe was in the range 40–135 pM. The SMRT link software, a PacBio web-based end-to-end workflow manager, was used to set-up and monitor the run, as well as perform primary and secondary analysis of the data upon completion.

***Hi-C data***

For Hi-C library preparation, DNA was fragmented to a size of 400 to 600 bp using a Covaris E220 sonicator. The DNA was then enriched, barcoded, and amplified using the NEBNext Ultra II DNA Library Prep Kit following manufacturers’ instructions. The Hi-C sequencing was performed using paired-end sequencing with a read length of 150 bp on an Illumina NovaSeq 6000 instrument.

***RNA***

Poly(A) RNA-Seq libraries were constructed using the NEB Ultra II RNA Library Prep kit, following the manufacturer’s instructions. RNA sequencing was performed on the Illumina NovaSeq 6000 instrument.

### Genome assembly, curation and evaluation

***Assembly***

The HiFi reads were first assembled using Hifiasm ( Cheng *et al.*, 2021) with the --primary option. Haplotypic duplications were identified and removed using purge_dups ( Guan *et al.*, 2020). The Hi-C reads were mapped to the primary contigs using bwa-mem2 ( Vasimuddin *et al.*, 2019). The contigs were further scaffolded using the provided Hi-C data ( Rao *et al.*, 2014) in YaHS ( Zhou *et al.*, 2023) using the --break option for handling potential misassemblies. The scaffolded assemblies were evaluated using Gfastats ( Formenti *et al.*, 2022), BUSCO ( Manni *et al.*, 2021) and MERQURY.FK ( Rhie *et al.*, 2020).

The mitochondrial genome was assembled using MitoHiFi ( Uliano-Silva *et al.*, 2023), which runs MitoFinder ( Allio *et al.*, 2020) and uses these annotations to select the final mitochondrial contig and to ensure the general quality of the sequence.

***Assembly curation***

The assembly was decontaminated using the Assembly Screen for Cobionts and Contaminants (ASCC) pipeline (article in preparation). Flat files and maps used in curation were generated in TreeVal ( Pointon *et al.*, 2023). Manual curation was primarily conducted using PretextView ( Harry, 2022), with additional insights provided by JBrowse2 ( Diesh *et al.*, 2023) and HiGlass ( Kerpedjiev *et al.*, 2018). Scaffolds were visually inspected and corrected as described by Howe *et al*. (2021). Any identified contamination, missed joins, and mis-joins were corrected, and duplicate sequences were tagged and removed. The sex chromosome was identified by synteny analysis. The curation process is documented at https://gitlab.com/wtsi-grit/rapid-curation (article in preparation).

***Assembly quality assessment***

The Merqury.FK tool ( Rhie *et al.*, 2020) was used to evaluate *k*-mer completeness and assembly quality for the primary and alternate haplotypes using the *k*-mer databases ( *k* = 31) that were pre-computed prior to genome assembly. The analysis outputs included assembly QV scores and completeness statistics.

A Hi-C contact map was produced for the final, public version of the assembly. The Hi-C reads were aligned using bwa-mem2 ( Vasimuddin *et al.*, 2019) and the alignment files were combined using SAMtools ( Danecek *et al.*, 2021). The Hi-C alignments were converted into a contact map using BEDTools ( Quinlan & Hall, 2010) and the Cooler tool suite ( Abdennur & Mirny, 2020). The contact map is visualised in HiGlass ( Kerpedjiev *et al.*, 2018).

The genome was analysed within the BlobToolKit environment ( Challis *et al.*, 2020) and BUSCO scores ( Manni *et al.*, 2021) were calculated.

Table 4 contains a list of relevant software tool versions and sources.

**Table 4.** Software tools: versions and sources.

Software tool	Version	Source
BEDTools	2.30.0	https://github.com/arq5x/bedtools2
BLAST	2.14.0	ftp://ftp.ncbi.nlm.nih.gov/blast/executables/blast+/
BlobToolKit	4.3.7	https://github.com/blobtoolkit/blobtoolkit
BUSCO	5.4.3 and 5.5.0	https://gitlab.com/ezlab/busco
bwa-mem2	2.2.1	https://github.com/bwa-mem2/bwa-mem2
Cooler	0.8.11	https://github.com/open2c/cooler
DIAMOND	2.1.8	https://github.com/bbuchfink/diamond
fasta_windows	0.2.4	https://github.com/tolkit/fasta_windows
FastK	427104ea91c78c3b8b8b49f1a 7d6bbeaa869ba1c	https://github.com/thegenemyers/FASTK
Gfastats	1.3.6	https://github.com/vgl-hub/gfastats
GoaT CLI	0.2.5	https://github.com/genomehubs/goat-cli
Hifiasm	0.19.8-r587	https://github.com/chhylp123/hifiasm
HiGlass	44086069ee7d4d3f6f3f001256 9789ec138f42b84aa44357826 c0b6753eb28de	https://github.com/higlass/higlass
Merqury.FK	d00d98157618f4e8d1a919002 6b19b471055b22e	https://github.com/thegenemyers/MERQURY.FK
MitoHiFi	3	https://github.com/marcelauliano/MitoHiFi
MultiQC	1.14, 1.17, and 1.18	https://github.com/MultiQC/MultiQC
NCBI Datasets	15.12.0	https://github.com/ncbi/datasets
Nextflow	23.04.0-5857	https://github.com/nextflow-io/nextflow
PretextView	0.2.5	https://github.com/sanger-tol/PretextView
purge_dups	1.2.5	https://github.com/dfguan/purge_dups
samtools	1.16.1, 1.17, and 1.18	https://github.com/samtools/samtools
sanger-tol/ascc	-	https://github.com/sanger-tol/ascc
sanger-tol/blobtoolkit	0.6.0	https://github.com/sanger-tol/blobtoolkit
Seqtk	1.3	https://github.com/lh3/seqtk
Singularity	3.9.0	https://github.com/sylabs/singularity
TreeVal	1.0.0	https://github.com/sanger-tol/treeval
YaHS	1.2a.2	https://github.com/c-zhou/yahs

### Genome annotation

The Ensembl Genebuild annotation system ( Aken *et al.*, 2016) was used to generate annotation for the *Perizoma flavofasciatum* assembly (GCA_958496245.1) in Ensembl Rapid Release at the EBI. Annotation was created primarily through alignment of transcriptomic data to the genome, with gap filling via protein-to-genome alignments of a select set of proteins from UniProt ( UniProt Consortium, 2019).

### Wellcome Sanger Institute – Legal and Governance

The materials that have contributed to this genome note have been supplied by a Darwin Tree of Life Partner. The submission of materials by a Darwin Tree of Life Partner is subject to the **‘Darwin Tree of Life Project Sampling Code of Practice’**, which can be found in full on the Darwin Tree of Life website here. By agreeing with and signing up to the Sampling Code of Practice, the Darwin Tree of Life Partner agrees they will meet the legal and ethical requirements and standards set out within this document in respect of all samples acquired for, and supplied to, the Darwin Tree of Life Project.

Further, the Wellcome Sanger Institute employs a process whereby due diligence is carried out proportionate to the nature of the materials themselves, and the circumstances under which they have been/are to be collected and provided for use. The purpose of this is to address and mitigate any potential legal and/or ethical implications of receipt and use of the materials as part of the research project, and to ensure that in doing so we align with best practice wherever possible. The overarching areas of consideration are:

•   Ethical review of provenance and sourcing of the material

•   Legality of collection, transfer and use (national and international)

Each transfer of samples is further undertaken according to a Research Collaboration Agreement or Material Transfer Agreement entered into by the Darwin Tree of Life Partner, Genome Research Limited (operating as the Wellcome Sanger Institute), and in some circumstances other Darwin Tree of Life collaborators.

## Data Availability

European Nucleotide Archive: Perizoma flavofasciatum (sandy carpet). Accession number PRJEB63430; https://identifiers.org/ena.embl/PRJEB63430. The genome sequence is released openly for reuse. The *Perizoma flavofasciatum* genome sequencing initiative is part of the Darwin Tree of Life (DToL) project. All raw sequence data and the assembly have been deposited in INSDC databases. Raw data and assembly accession identifiers are reported in Table 1 and Table 2.

## Author information

Members of the University of Oxford and Wytham Woods Genome Acquisition Lab are listed here: https://doi.org/10.5281/zenodo.12157525.

Members of the Darwin Tree of Life Barcoding collective are listed here: https://doi.org/10.5281/zenodo.12158331.

Members of the Wellcome Sanger Institute Tree of Life Management, Samples and Laboratory team are listed here: https://doi.org/10.5281/zenodo.12162482.

Members of Wellcome Sanger Institute Scientific Operations: Sequencing Operations are listed here: https://doi.org/10.5281/zenodo.12165051.

Members of the Wellcome Sanger Institute Tree of Life Core Informatics team are listed here: https://doi.org/10.5281/zenodo.12160324.

Members of the Tree of Life Core Informatics collective are listed here: https://doi.org/10.5281/zenodo.12205391.

Members of the Darwin Tree of Life Consortium are listed here: https://doi.org/10.5281/zenodo.4783558.
