# Supplementary material for: The genome sequence of the Sandy Carpet moth, Perizoma flavofasciatum (Thunberg, 1792)
Source: Wellcome Open Res. 2025 Feb 3;10:40. [Version 1] doi: 10.12688/wellcomeopenres.23612.1 (PMC11889404; doi:10.12688/wellcomeopenres.23612.1)
Supplement: Peer review history

# Reviewer report: Reviewer response for version 1

**DOI:** 10.21956/wellcomeopenres.26048.r118672

**Published:** 2025-03-07

**Authors:** Sivasankaran Kuppusamy

**Affiliations:** 1 Entomology Research Institute, Loyola College, Chennai, India

Authors have produced a comprehensive chromosome-level genome assembly of *Perizoma flavofasciatum *(Thunberg, 1792). The genome sequence scaffolded into chromosomes through the assembly. Through the annotation, a total of 11,915 protein-coding genes, 1605 non-coding genes and 22,268 gene transcripts were detected. 

**Minor comments on the manuscript:**

- The authors have given the full name of the genus, *Perizoma flavofasciatum*, throughout the text. If at all possible, start with the full genus name and then abbreviate it to *P. flavofasciatum.*
-  In the Background third paragraph last line authors have mentioned within the parenthesis ( *Silene spp.*). “spp” may not be in italics. 

Above all, I confirm that the manuscript meets the necessary scientific standard and is suitable for indexing.

Are sufficient details of methods and materials provided to allow replication by others?

Yes

Is the rationale for creating the dataset(s) clearly described?

Yes

Are the datasets clearly presented in a useable and accessible format?

Yes

Are the protocols appropriate and is the work technically sound?

Yes

Reviewer Expertise:

Phylogenetic analysis of Noctuoidea moths using mitogenome sequence

I confirm that I have read this submission and believe that I have an appropriate level of expertise to confirm that it is of an acceptable scientific standard.

# Reviewer report: Reviewer response for version 1

**DOI:** 10.21956/wellcomeopenres.26048.r118668

**Published:** 2025-03-04

**Authors:** Jeffrey Marcus

**Affiliations:** 1 University of Manitoba, Winnipeg, Manitoba, Canada

In this manuscript, the authors describe the sequencing and assembly of the *Perizoma “flavofasciatum” *genome using DNA from two adult male specimens collected in the UK. The primary genome sequence assembly includes proposed chromosomal pseudomolecule sequences for 29 autosomes, the Z sex chromosome, and a complete mitochondrial genome of 16,613 bp. Gene annotation of the assembly identified 11,915 putative protein-coding genes and 1,605 non-coding genes. On the whole, this is a useful contribution to the scientific literature, but please see my comments below:

 Some suggestions to the authors:

1. The authors identify the species whose genome is being analyzed (in the title and throughout the text) as *Perizoma “flavofasciatum” *(Thunberg, 1792) which I believe is not written correctly.  The correct species name is *Perizoma flavofasciata *(Thunberg, 1792).
2. Method of Specimen identification: The individual who did the specimen identification was named, but keys/species descriptions consulted, or the morphological characters used for the identification have not been included in the manuscript, unless the morphological details included in the background section were used for this purpose (it’s not clear from the text).  I have made similar suggestions in previous reviews for this journal:

Marcus J. Peer Review Report For: The genome sequence of the Poplar Grey moth, *Subacronicta megacephala *(Denis & Schiffermüller, 1775) [version 1; peer review: 1 approved, 2 approved with reservations]. Wellcome Open Res 2024, **9**:696. doi: 10.21956/wellcomeopenres.25766.r116100

 Marcus J. 2024. Peer Review Report For: The genome sequence of a tortricid moth, *Lathronympha strigana *(Fabricius, 1775) [version 1; peer review: 1 approved, 1 approved with reservations]. Wellcome Open Res 2023, **8**:335. doi: 10.21956/wellcomeopenres.21965.

 Kopchak, N. and J. M. Marcus 2004. Peer Review Report For: The genome sequence of the White-barred Knot-horn, *Elegia similella *(Zincken, 1818). [version 1; peer review: 1 approved with reservations]. Wellcome Open Res 2024, **9**:186. doi: 10.21956/wellcomeopenres.23460.r100551

 Marcus J.M. 2024. Peer Review Report For: The genome sequence of the bramble shoot moth, *Notocelia uddmanniana* (Linnaeus, 1758) [version 1; peer review: 1 approved with reservations]. Wellcome Open Res 2021, **6**:348. doi: 10.21956/wellcomeopenres.19338.r79177

 Marcus J.M. 2024. Peer Review Report For: The genome sequence of the March moth, *Alsophila aescularia *(Denis & Schiffermüller) [version 1; peer review: 2 approved with reservations]. Wellcome Open Res 2024, **9**:50. doi: 10.21956/wellcomeopenres.22854.r86299

 I feel strongly that such details of how morphological identifications were conducted should be a requirement for publication.

 3. The authors report that the specimens used for this study were collected and identified by Douglas Boyes and Jo Davis.  There is a similarity between these names and the names of the first two authors of this paper, but it is not clear if “Jonathan Davis” and “Jo Davis” are the same person.  I suggest that if they are one and the same, that the same first name be used in the text as in the author list.

 4. Methods:  last line of the first paragraph:  there appears to be a word missing in the phrase “…identified by Douglas Boyes (University of Oxford) and on dry ice.”  Perhaps “stored on dry ice” is what was intended?

 5. Assembly of the mitochondrial genome:   The *Perizoma* mitochondrial genome (OY292449.1) is reported to be 16,613 bp long which makes it very atypical of Lepidoptera and of insects more generally.  Most Lepidoptera have mitochondrial genomes between 15,200 and 15,400 bp.  Much larger mitochondrial genome sizes are energetically very unfavorable (due to the additional energy required to replicate the DNA of a larger genome) and would be expected to experience strong negative selection. My alignment of this newly reported, but unannotated *Perizoma *mitochondrial genome with other previously annotated mitogenomes (not shown) from the Geometridae suggests that if indeed there is an assembly problem, it is in the control region, as the remainder of the mitogenome sequence maps well onto the sequences of other geometrid species and shows synteny.  Using BLAST to compare OY292449.1 with the GenBank database recovers itself as the best match (as expected), but then the next 14 BLAST matches each show 95% (1/14) or 100% (13/14) query coverage.  All 14 of these matches (OZ224322.1, OZ076559.1, OZ197163.1, OZ234991.1, OZ205108.1, OY560209.1, OZ076728.1, OZ203711.1, OX596166.1, OZ205075.1, OX388289.1, OY757060.1, OY292480.1, OZ205568.1) are roughly 10% larger in mitogenome size than expected, all were generated by the Tree of Live Consortium, and all were almost certainly assembled using the same MitoHiFi and Mitofinder algorithms used by the authors of the current work.  I am concerned that a systematic error is being made that is compromising the validity of the mitochondrial genome assemblies from multiple species.  I could be convinced about the correctness of the *Perizoma* mitochondrial genome assembly (OY292449.1) if the authors are able to produce the identical assembly from their data set using software with a different computational approach.  One such alternative approach, assembling mitogenomes using a reference sequence is described here:

 Marcus, J. M. 2018. Our love-hate relationship with DNA barcodes, the Y2K problem, and the search for next generation barcodes. AIMS Genetics **5 **(1): 1–23. doi: 10.3934/genet.2018.1.1

 If they choose to confirm their result in this way, I recommend that they use Genbank mitogenome accession KP027400.1 from *Operophtera brumata* (Derks et al. 2015) as the reference sequence, as this sequence is close to the expected typical mitogenome size and is annotated reasonably well.

 6. For future work, I suggest that the researchers preferentially sequence specimens from the heterogametic sex when assembling genomes for previously unstudied species (in the case of Lepidoptera, the heterogametic sex is female), so that draft sequence assembles can be prepared for both sex chromosomes.

Are sufficient details of methods and materials provided to allow replication by others?

Partly

Is the rationale for creating the dataset(s) clearly described?

Yes

Are the datasets clearly presented in a useable and accessible format?

Yes

Are the protocols appropriate and is the work technically sound?

Partly

Reviewer Expertise:

Evolutionary biology of insects, phylogenomics

I confirm that I have read this submission and believe that I have an appropriate level of expertise to confirm that it is of an acceptable scientific standard, however I have significant reservations, as outlined above.

## References

[1] : The Genome of Winter Moth (Operophtera brumata) Provides a Genomic Perspective on Sexual Dimorphism and Phenology. *Genome Biol Evol* .2015;7(8) : 10.1093/gbe/evv145 2321-32 10.1093/gbe/evv145 26227816 PMC4558862

# Reviewer report: Reviewer response for version 1

**DOI:** 10.21956/wellcomeopenres.26048.r118663

**Published:** 2025-02-24

**Authors:** Carlos Lopez-Vaamonde

**Affiliations:** 1 Unité Zoologie Forestière, INRAE Centre Val de Loire, Orleans, France

The assembly quality value, k-mer completeness, BUSCO completeness score, and the percentage of the assembly assigned to chromosomes all indicate a high-quality assembly with significant coverage and completeness.

 Please find below a few minor comments for the authors to consider:

1. **Title**: Please include the order and family (Lepidoptera, Geometridae) after the species name.
2. **Methods**: You state, *“Tissue from the whole organism was homogenized.”* Does this mean the wings were not preserved as a voucher? If so, I suggest clarifying: *“… including the wings.”*
3. **DNA Barcode & Metadata**: I strongly recommend depositing the DNA barcode and all associated metadata in the BOLD (Barcode of Life Data) system. Please ensure that all relevant details are included, such as the identifier of the specimen. Additionally, it would be valuable to verify whether the barcode of the voucher clusters within the same Barcode Index Number (BIN) as other *Perizoma flavofasciata* sequences, confirming its lineage:
BOLD Barcode Cluster

Are sufficient details of methods and materials provided to allow replication by others?

Yes

Is the rationale for creating the dataset(s) clearly described?

Yes

Are the datasets clearly presented in a useable and accessible format?

Yes

Are the protocols appropriate and is the work technically sound?

Yes

Reviewer Expertise:

NA

I confirm that I have read this submission and believe that I have an appropriate level of expertise to confirm that it is of an acceptable scientific standard.
